# Abrogation of HLA surface expression using CRISPR/Cas9 genome editing: a step toward universal T cell therapy

**DOI:** 10.1038/s41598-020-74772-9

**Published:** 2020-10-20

**Authors:** Jeewon Lee, Joong Hyuk Sheen, Okjae Lim, Yunjung Lee, Jihye Ryu, Duckhyang Shin, Yu Young Kim, Munkyung Kim

**Affiliations:** MOGAM Institute for Biomedical Research, 93, 30beon-gil, Ihyeon-ro, Giheung-gu, Yongin-si, Gyeonggi-do 16924 South Korea

**Keywords:** Cell biology, Immunology, Molecular biology

## Abstract

As recent advancements in the chimeric antigen receptor-T cells have revolutionized the way blood cancers are handled, potential benefits from producing off-the-shelf, standardized immune cells entail the need for development of allogeneic immune cell therapy. However, host rejection driven by HLA disparity in adoptively transferred allogeneic T cells remains a key obstacle to the universal donor T cell therapy. To evade donor HLA-mediated immune rejection, we attempted to eliminate T cell’s HLA through the CRISPR/Cas9 gene editing system. First, we screened 60 gRNAs targeting B2M and multiple sets of gRNA each targeting α chains of HLA-II (DPA, DQA and DRA, respectively) using web-based design tools, and identified specific gRNA sequences highly efficient for target deletion without carrying off-target effects. Multiplex genome editing of primary human T cells achieved by the newly discovered gRNAs yielded HLA-I- or HLA-I/II-deficient T cells that were phenotypically unaltered and functionally intact. The overnight mixed lymphocyte reactions demonstrated the HLA-I-negative cells induced decreased production of IFN-γ and TNF-α in alloreactive T cells, and deficiency of HLA-I/II in T cells further dampened the inflammatory responses. Taken together, our approach will provide an efficacious pathway toward the universal donor cell generation by manipulating HLA expression in therapeutic T cells.

## Introduction

The emergence of chimeric antigen receptor (CAR)-T cell therapy has changed the paradigm of cancer immunotherapy by virtue of its durable remission with manageable toxicity profile. Two CD19-targeting autologous CAR-T products have shown high rates of responses in clinical studies and recently received FDA approval for the treatment of B-cell lymphoid malignancies^1–3^. However, challenges in the utilization of autologous cells from patients extend from the labor-intensive, high-cost nature of the manufacturing procedures required for individualized therapy to the limited quantity and/or sub-optimal intrinsic quality of patient T cells. Genomic, phenotypic, and functional analyses of CD19 CAR-T cells from treatment-responding patients in chronic lymphocytic leukemia demonstrated that the intrinsic properties of T cells, such as upregulation of IL-6/STAT3 signaling and enhanced transcription of memory T cell-related genes, are key determinants of the efficacy of the therapy^4^. Limited efficacy associated with epigenetic modulation, impaired functionality, and more exhaustion and apoptotic phenotypes of the infused autologous T cell products can preclude durable remission and clinical response following treatment^4–7^. Furthermore, a recent report in a trial of autologous CAR-T cell therapy documented the unintentional transduction of a single leukemic B cell with anti-CD19 CAR during manufacturing and its product masking the target antigen, thereby escaping CAR-T recognition and resisting the therapy^8^. These limitations could be overcome by employing allogeneic T cells obtained from “fitted” healthy donors as a source of universal CAR-T cell production. But issues related to HLA barriers, such as the risks of graft-versus host disease (GvHD), as well as host-mediated rejection of the infused allogeneic cells, are the major obstacles to this intervention.

Donor allogeneic T cells recognize the “non-self” antigens of the host cells and elicit GvHD by TCR αβ-mediated signaling^9^. Elimination of endogenous TCR from the donor T cells by disruption of the TCR α or β chain using different gene editing technologies, such as zinc finger nucleases (ZFNs), transcription activator-like effector nucleases (TALENs), or the CRISPR/Cas9 system, has been shown to prevent alloresponses from donor T cells^10–14^. Using γδ T cells, which are unlikely to cause GvHD, to generate CAR-T cells is an alternative approach without genetic manipulation to circumvent GvHD^15^. Conversely, graft rejection is caused by the host immune system recognizing the mismatching HLAs of the infused or transplanted allogeneic cells. One of the approaches to evade host-mediated rejection is to eliminate HLA-I from donor T cells. Ren et al*.* demonstrated deletion of the B2M gene, an essential component for the cell surface expression of HLA-I, through the CRISPR/Cas9 system in human T cells and showed that HLA-I-deficient T cells resulted in a reduction of surveillance of allogeneic T cells compared to non-gene-edited T cells^13^. Reports by others further indicated that HLA-II molecules are highly expressed on activated T cells as well^16,17^, and HLA-II mismatch can activate the alloreactive CD4^+^ T cells of the recipient^18,19^. While editing of multiple HLA targets in allogeneic T cells may offer great potential for universal CAR-T cell therapy, no report has yet demonstrated highly efficient methods to simultaneously abrogate the expression of HLA class I and II in T cells and its impact in inducing alloresponse.

In this study, we discovered gRNAs specific to B2M and HLA class II that enabled highly efficient and on-target genome editing, and delivered those gRNAs simultaneously into primary human T cells. We have targeted α chains of HLA-II genes (HLA-DRA, DQA and DPA) because they are relatively less polymorphic compared with β chains. The gene-edited HLA-I/II-negative T cells retained their T cell functionality and phenotypes upon in vitro stimulation. Additionally, in vitro mixed lymphocyte reactions revealed that alloresponses in responder cells were dampened against HLA-I/II-negative T cells compared to HLA-I-negative cells, solidifying a conceptual framework that narrates the role of HLA-II plays in infused, therapeutic allogeneic cells during host-mediated rejection.

## Results

### Newly discovered gRNAs for HLA deletion showed high deletion efficiency without off-target effects

**Figure 1:**
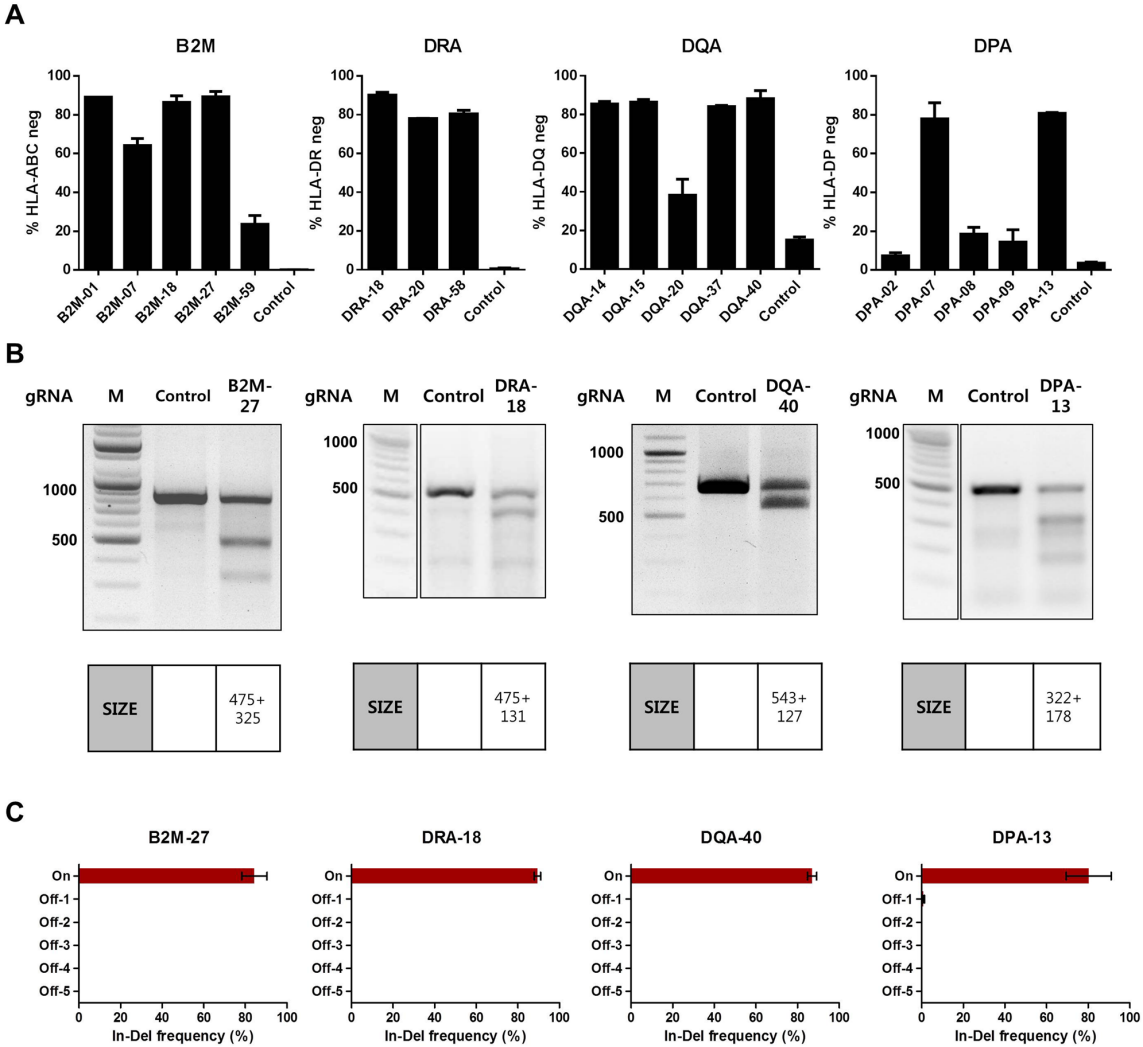
Screening of gRNAs targeting B2M or α chains of HLA-II genes. Raji cells were transfected with the indicated gRNAs complexed with Cas9 protein (gRNA/Cas9) and cultured for 7 days. (**A)** Target deletion efficiency of the indicated gRNAs as detected by flow cytometry in Raji cells. Percentages of target HLA-negative cells out of total cells are presented as % HLA neg (n = 3). Error bars indicate mean ± standard error of the mean (SEM). (**B**) Amount of HLA-targeted gene disruption measured by a mismatch-sensitive enzyme assay on DNA amplified from target-gRNA/Cas9- or non-targeting gRNA/Cas9-transfected Raji cells. Expected sizes of the DNA fragments are specified in separate tables below. Representative images are shown from three independent experiments. Full-length gels are presented in Supplementary Figure S3. M, DNA Marker. (**C**) On-target and off-target mutagenesis measured by NGS on genomic DNA amplified from indicated gRNA/Cas9-transfected Raji cells (n = 3). Error bars indicate mean ± SEM. On, on-target. Off, off-target.

In order to ablate the expression of HLA-I and HLA-II on the cell surface, we attempted to target genes encoding β_2_-microglobulin (B2M) and α chains of HLA-II molecules with the CRISPR/Cas9 gene editing system. We employed web-based gRNA designing tools such as CHOPCHOP^20^, E-CRISP^21^ and CRISPR-ERP^22^ to identify gRNA sequences targeting the B2M, HLA-DRA, HLA-DQA and HLA-DPA gene. Out of hundreds of gRNA candidate sequences per target, we narrowed the lists to 60 gRNA sequences for each target gene (see the Methods section for the criteria and details). Then, the gRNAs were transcribed in vitro and transfected into Raji cells together with the Cas9 protein to validate their target deletion efficiency. 20 gRNAs were tested per experiment, and the gRNAs showing deletion efficiencies exceeding the internal criteria in three independent experiments were selected (Supplementary figure S1). The selected gRNAs were tested again collectively in a single experiment, and the gRNAs highly efficient in target deletion (> 70%) were identified (Fig. 1A). Currently, 29 DRA alleles, 216 DQA1 alleles and 161 DPA1 alleles are assigned in the IPD-IMGT/HLA database^23^, and genomic sequences are available for 28 DRA alleles, 140 DQA1 alleles and 86 DPA1 alleles^23^ (Supplementary table S1). Among the newly identified gRNAs, DQA-40 and DPA-13 gRNA were ultimately selected for further experiments because their target sequences are conserved in all DQA1 or DPA1 alleles whose sequences are publicly available. All known DRA allele sequences were covered by the three selected gRNAs above, so we chose DRA-18 gRNA as a final candidate because it had the highest deletion efficiency.

Genomic mutations in each target were confirmed by mismatch-sensitive enzyme-based assays and deep sequencing (Fig. 1B,C). To analyze possible off-target effects, we sequenced the top five predicted off-target sites for each target after gRNA delivery and observed no detectable off-target events (Fig. 1C).

### CRISPR-Cas9-mediated multiplex genome editing efficiently ablates expression of HLA on human primary T cells without affecting effector function

While HLA-I is expressed by all nucleated cells, constitutive HLA-II expression is conventionally thought to be restricted to professional antigen-presenting cells such as dendritic cells, B cells and macrophages. However, previous reports by others as well as our own data showed increased levels of HLA-II in NK cells or T cells when they were expanded or activated ex vivo^16,24^ (Supplementary figure S2), and host-derived alloreactive immune rejection is mediated by expression of HLA-I and HLA-II molecules on infused third-party cells.

**Figure 2:**
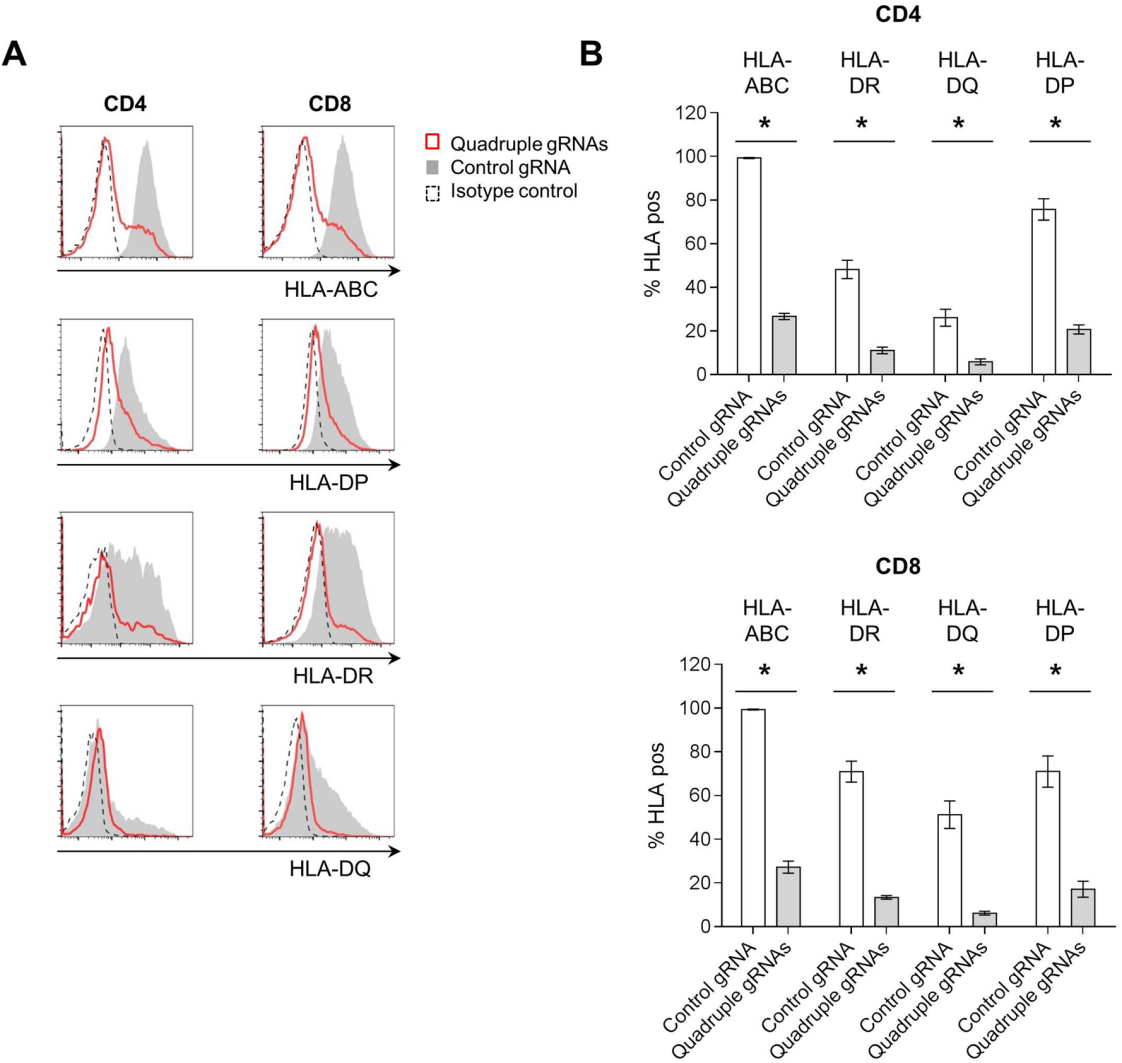
Efficient HLA disruption in human T cells by multiplex genome editing. Primary human CD3^+^ T cells were transfected with a mixture of gRNAs targeting B2M, HLA-DRA, HLA-DQA and HLA-DPA (Quadruple gRNAs) or non-targeting gRNA (control gRNA) together with Cas9 protein and expanded for 13 days ex vivo. (**A**) Representative histograms of HLA expression on CD4^+^ (left) or CD8^+^ (right) T cells gated from 13-day expanded CD3^+^ T cells. (**B**) Quantification of HLA expression in CD4^+^ (top) and CD8^+^ (bottom) T cells in (**A**) (pooled results from three independently performed experiments, total n = 6). Percentages of target HLA-positive cells out of total cells are presented as % HLA pos. Error bars indicate mean ± SEM. *, *p* = 0.0313, by Wilcoxon matched-pairs signed rank test.

**Figure 3:**
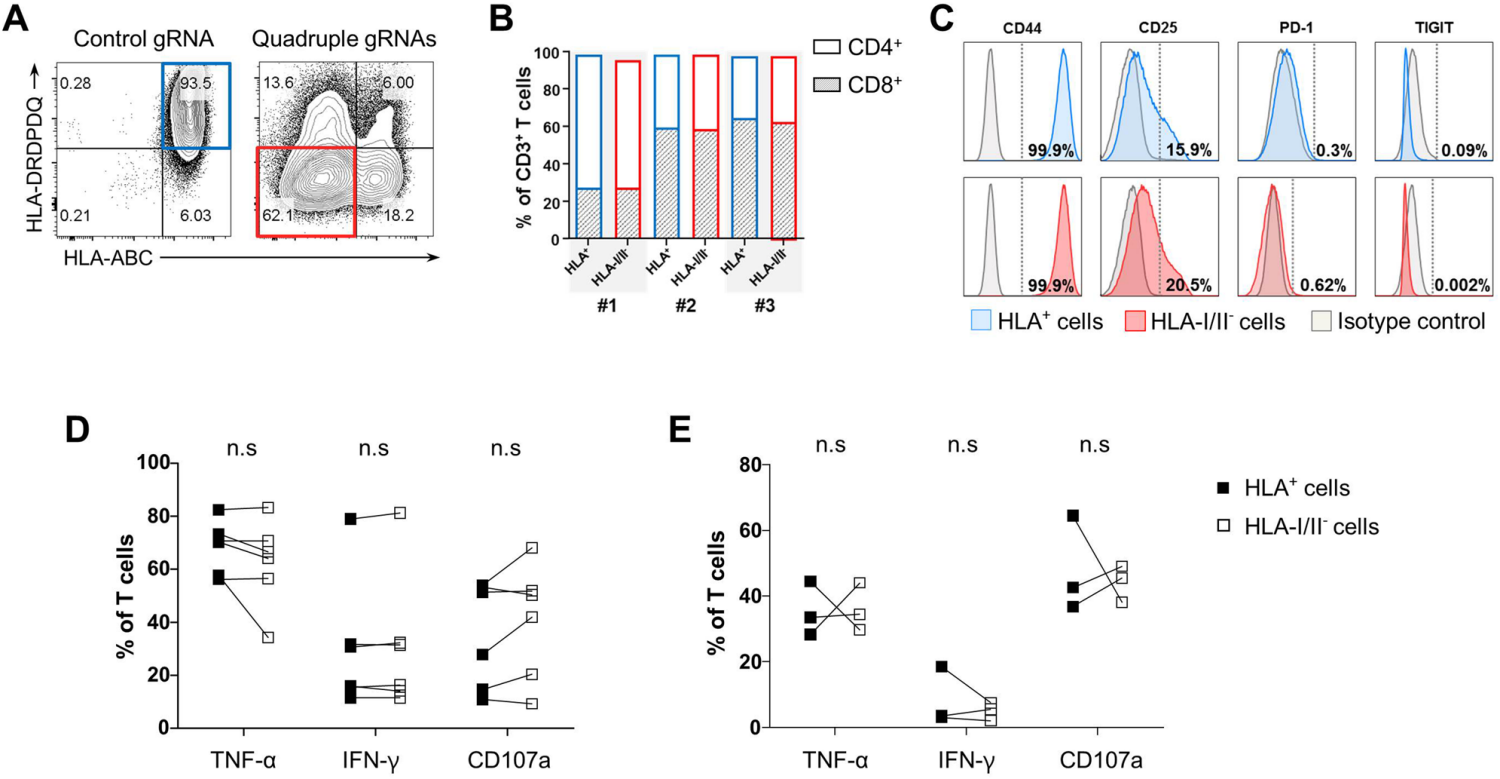
HLA-I/II-negative human T cells retain functional and phenotypic properties. Primary CD3^+^ T cells were transfected with a mixture of gRNAs targeting B2M, HLA-DRA, HLA-DQA and HLA-DPA (Quadruple gRNAs) or non-targeting gRNA (control gRNA) together with Cas9 protein and expanded for 14 days ex vivo. (**A**) A flow cytometry image of HLA ablation in CD3^+^ T cells transfected with HLA gRNAs. A representative plot is shown from five different donors. Boxes on the plots indicate the HLA-positive control T cells (blue) or HLA-I/II-negative T cells (red) analyzed in (**B**–**E**). (**B**) Distribution of CD4^+^ and CD8^+^ T cells in HLA positive control T cells or HLA-I/II-negative T cells gated from (**A**). Results from three different donors are shown. (**C**) Histograms depicting expression of activation or exhaustion markers in HLA-positive control T cells (top) or HLA-I/II-negative T cells (bottom) gated from (**A**). A representative image from five different donors is shown. (**D**,**E**) Secretion of TNF-α, IFN-γ and CD107a release were measured by intracellular staining in HLA-positive control T cells or HLA-I/II-negative T cells gated from (**A**). Cells were stimulated with PMA/ionomycin (**D**) or Dynabeads Human T-Activator CD3/CD28 (**E**). Results from six (**D**) or three (**E**) different PBMC donors are shown. n.s, not significant (*p* > *0.05*) by Wilcoxon matched-pairs signed rank test.

In order to examine whether selected gRNAs can simultaneously suppress the expression of HLA-I and HLA-II in T cells, we transfected a mixture of four gRNAs each targeting the B2M, HLA-DRA, HLA-DQA and HLA-DPA gene (referred to hereafter as quadruple gRNAs) or non-targeting gRNA together with the Cas9 protein into primary human CD3^+^ T cells isolated from healthy donor-derived PBMCs. After 13 days of expansion of the transfected cells, the control CD4^+^ and CD8^+^ T cells treated with non-targeting gRNA expressed high levels of HLA-I and HLA-II, as previously reported, and this expression was efficiently downregulated by transfection with the quadruple gRNAs (Fig. 2A,B). The quadruple-gene-edited CD3^+^ T cells exhibited 62.1% of HLA-I/II-double-negative cells (Fig. 3A) and expanded 44-fold in 13 days of culture, which is one thirds of the un-transfected, control T cells (129-fold) (Supplementary figure S4). Reduction in viability observed after the gene editing (Supplementary figure S5) may suggest the transient genotoxicity from multiple DNA breaks, ultimately resulting in decreased expansion of the quadruple-gene-edited cells.

To test whether the HLA-negative T cells maintain the CD4/CD8 subset ratio and T cell phenotypes, we measured expression of surface CD4, CD8 and T cell activation/exhaustion markers in the HLA-negative T cells and control T cells. As seen in Fig. 3B,C, there were no differences in CD4^+^/CD8^+^ T cell distribution and phenotypes between the HLA-I/II-negative T cells and the control T cells.

To further analyze the effect of HLA ablation on T cell effector function, we measured proinflammatory cytokine production and CD107a release in the genetically engineered T cells stimulated with PMA/ionomycin or anti-CD3/CD28 beads. No significant changes were noticed in the production of TNF-α and IFN-γ or the release of cytotoxic granules between the HLA-I/II-negative T cells and control T cells in vitro (Fig. 3D,E). Taken together, our data demonstrate that simultaneous delivery of multiple gRNAs targeting B2M, HLA-DRA, HLA-DQA and HLA-DPA can efficiently eliminate HLA molecules from human primary T cells without affecting their effector functionality or surface phenotypes.

### Alloreactive T cell responses are mediated by expression of HLA-I/II in target T cells

**Figure 4:**
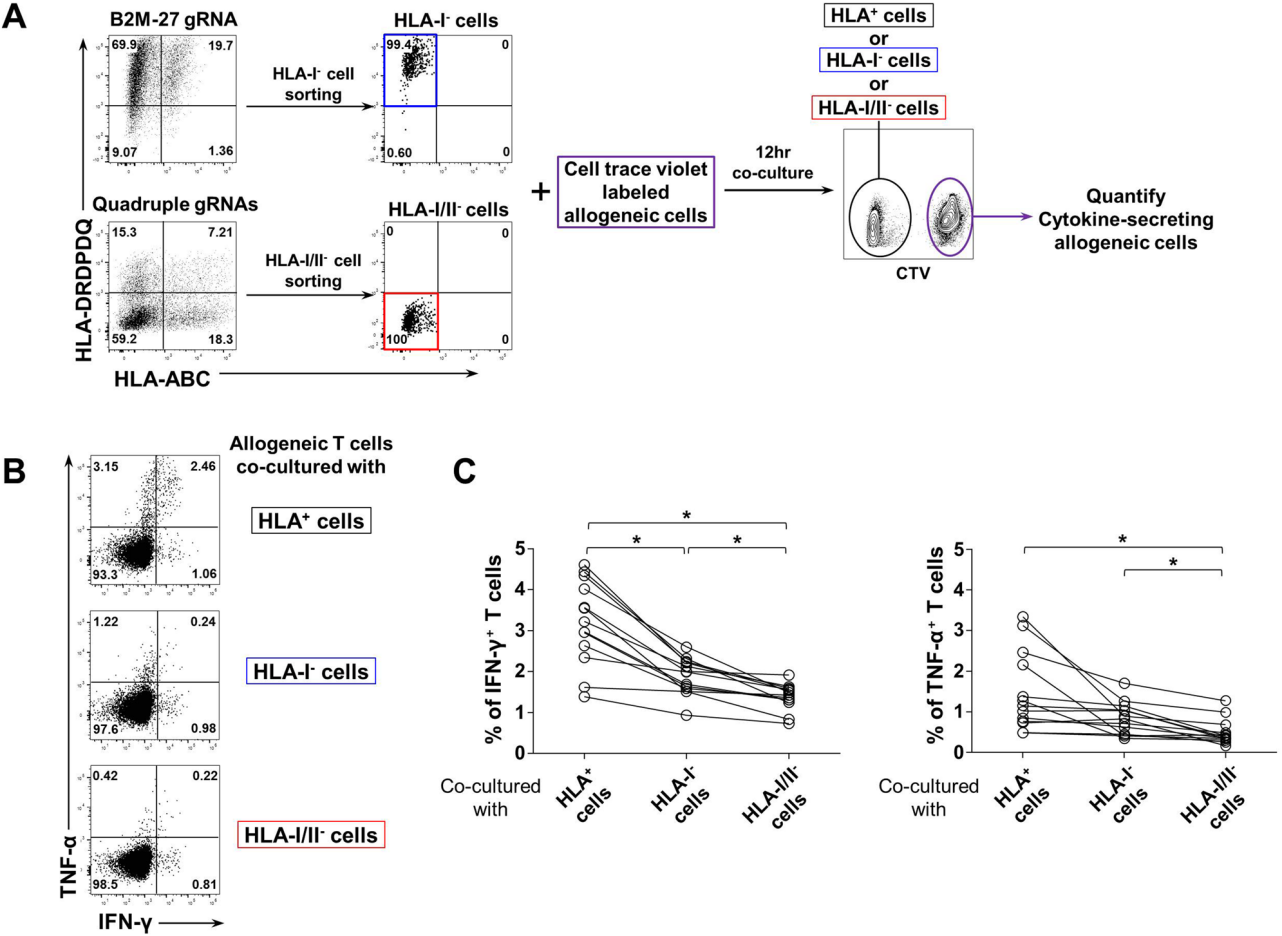
HLA-II ablation in HLA-I-negative T cells further reduces allogeneic T cell activation. (**A**) A schematic diagram of experimental design for (**B**,**C**) Human primary CD3^+^ T cells isolated from healthy donor PBMC were transfected with non-targeting gRNA (control gRNA), B2M gRNA (B2M-27 gRNA), or a mixture of quadruple gRNAs each targeting B2M, HLA-DRA, HLA-DQA and HLA-DPA (quadruple gRNAs) together with Cas9 protein and expanded ex vivo. On day 14, the expanded T cells were sorted according to their HLA expression and γ-irradiated. Another set of allogeneic human PBMCs stained with CTV was co-cultured with the sorted T cells for 12-h. (**B**) A representative flow cytometry plot depicting the numbers of cytokine-producing allogeneic CTV^+^ CD3^+^ T cells co-cultured with the HLA^+^ (top), HLA-I^−^ (middle), or HLA-I/II^−^ T cells (bottom), respectively. (**C**) Quantification of IFN-γ (left) or TNF-α-secreting (right) cells among the total allogeneic CD3^+^ T cells from (**B**) are presented. Each dot represents different alloreactive donor (n  = 13). Pooled results from 4 independently performed experiments are shown. *p < 0.05, by Friedman test followed by Dunn’s multiple comparisons test.

To investigate whether the deletion of HLA molecules in T cells can alleviate alloreactive immune responses, HLA-I/II-positive, HLA-I-negative or HLA-I/II-negative cells were sorted from the non-target gRNA, B2M gRNA or quadruple gRNAs-treated CD3^+^ T cells, respectively (Fig. 4A). Then, the sorted cells were γ-irradiated and co-cultured with another set of allogeneic PBMCs stained with a CellTrace Violet (CTV) dye. The level of alloreactivity was determined by intracellular cytokine production in CD3^+^ T cells gated from the CTV^+^ allogeneic PBMCs. Our data revealed the elimination of HLA-I on the target cell surface diminished IFN-γ and TNF-α secretion from the allogeneic T cells, and the alloresponse was further abrogated by HLA-I/II ablation (Fig. 4B,C). These results indicate that HLA expression serves as an indispensable immune modulator required for eliciting alloreactive T cell responses and that elimination of both the HLA-I and HLA-II molecules on donor cells is crucial to dampen host-mediated rejections in the setting of allogeneic cellular therapy.

## Discussion

The current CAR-T cell therapies approved for the treatment of hematological malignancies require genetic engineering of patient-derived T cells. There are issues related to the manufacturing processes and quantity of these T cells in a fraction of pediatric or intensively treated patients, especially due to the heavy lymphocytotoxic chemotherapies the patients often receive^1,25^. Additionally, an increasing amount of data supports that greater clinical efficacy of the CAR-T cell therapy is determined by intrinsic factors of the T cells, such as poly-functionality and memory-like phenotypes^4,6,26^. Off-the-shelf CAR-T cells produced from healthy allogeneic donor T cells with the “fittest” phenotypes can increase the likelihood of therapeutic response and overcome the barriers of the autologous CAR-T cell therapy. The allogeneic CAR-T cell therapy, in order to evaluate its feasibility and broader applicability, must primarily address the HLA barriers that mediate the frequency and magnitude of alloimmune responses driven by the host.

To bypass the recognition of foreign HLA molecules by the host cells, we have identified highly specific gRNA sequences for HLA class I/II deletion on the cell surface and generated T cells lacking surface HLA molecules through simultaneous quadruple genome editing. Our in vitro data suggest that the genetically modified T cells retained their functionality and immune phenotypes and that alloresponses against the genetically engineered T cells were markedly reduced compared with control cells. To our knowledge, this is the first report presenting engineered human T cells devoid of both HLA-I and HLA-II molecules. Previously, HLA-A-negative primary human T cells were generated by ZFNs targeting the HLA-A locus and evaded HLA-A2-restricted T cell recognition^27^. Also, HLA-I-deficient human primary T cells generated by B2M gene disruption using the CRISPR/Cas9 gene editing system were shown to reduce alloresponses compared with wild-type control cells^13^. While the expression of HLA-II molecules in T cells was induced after ex vivo expansion (Supplementary figure S2), elimination of HLA-II molecules from the surface of primary T cells has not yet been reported, probably due to the highly polymorphic nature of HLA-II genes. We identified gRNAs able to cover the majority of alleles of each HLA-II α chain gene (HLA-DRA, HLA-DQA and HLA-DPA, respectively), which are less polymorphic than β chains. Among the gRNAs selected by their high target editing efficiency in Raji cells, we could select one gRNA for each target (DRA-18, DQA-40, DPA-13) that could cover all the target alleles whose sequences are known to date^23^.

In B lymphoblastoid cell lines or untransformed human endothelial cells, ablation of HLA-II was achieved by a single-gRNA-driven triple-gene knockout of the HLA class II β chain^28^ or by targeted disruption of class II transactivator (CIITA), an essential transcription factor for HLA-II genes^19,29^. Recently, several reports have described induced pluripotent stem cells (iPSCs) lacking HLA-II molecules generated by CRISPR/Cas9-mediated CIITA targeting^30–32^. Because the purpose of the above studies was to generate HLA-II knockout cells, which could be obtained by expanding a single engineered clone, the genome editing efficiency was of less importance. Indeed, the efficiency of HLA-II deletion before cell sorting or antibiotic selection was either low^28^ (shown by 1.5 to 9.7% of HLA-DR negative cells) or not specified^19,29–32^. We have tested the CIITA-targeting gRNAs used by the two studies above^29,31^, and obtained much lower efficiency in HLA-II ablation compared with HLA-II α chain gene-targeting gRNAs we have employed (Supplementary figure S6). In generating engineered mature primary cells, higher genome editing efficiency is critical due to the limited expansion potential of primary cells in the culture, implicating potential benefits provided by our gRNA sequences. Additionally, targeting transcription factors may confer unpremeditated transcriptional changes to several genes outside of HLA-II biology. For instance, expression of RAB4B, a protein involved in endocytic recycling, is augmented by CIITA, whereas *IL4*, *FasL*, and *COL1A2* are repressive targets of CIITA^33–36^. These findings imply that CIITA could play a broader role inside and outside of HLA-II-mediated antigen presentation processes and that targeting such a transcription factor may appeal less attractive for primary cell engineering aimed for clinical use.

Several issues still remain to be addressed with allogeneic HLA-negative T cells for therapeutic application. The first concern is that a lack of HLA class I/II molecules on the cell surface can induce the “missing-self” response from host NK cells, resulting in the lysis of the infused cells. To this end, HLA-E or HLA-G, a ligand for the NK inhibitory receptor CD94/NKG2A or ILT2, respectively, can be expressed on the HLA-negative donor cells to mitigate NK cell-mediated cytotoxicity^37,38^. Overexpression of other molecules interacting with NK inhibitory receptors^39,40^ or CD47, which transmits a “don’t-eat-me” signal and inhibits phagocytosis^30,41^, can be alternative targets to evade NK cell-driven host innate immune responses.

Another concern is off-target nuclease activity driven by highly efficient CRISPR/Cas9-mediated genome editing. Although we have demonstrated that the top five off-target site candidates for each target gene did not exhibit any mutations after transfection, it is worth noting that there could be off-target mutagenesis at sites other than predicted DNA sequences in a given genome, especially one with less than three mismatches. Further investigation is required to evaluate the unbiased identification of off-target cutting using whole-genome sequencing technology such as Digenome-seq^42^. Two Cas9 mutants, eSpCas9 and SpCas9-HF1, have demonstrated improved DNA targeting specificity^43,44^, and those variants could also be employed to avoid any undesirable off-target events if detected.

A recent paper describing multiple-gene-edited CAR-T cells by three gRNAs demonstrated the presence of chromosomal translocation during cell manufacturing^45^. In accordance with this result, our end-point PCR results using genomic DNA extracted from quadruple-gene-edited T cells from 3 different PBMC donors show that 19 rearrangements were induced among 20 potential rearrangements upon transfection with our four gRNAs (Supplementary figure S7). Most of the detected rearrangements were decreased during the expansion while only one translocation (DQA2:DRA) were slightly increased in 2 donors on expansion day 28. Together with the previous data illustrating declined proliferative capacity of the multiple-gRNA-transfected T cells compared with the controls (Supplementary figure S4), these results implicate that evident chromosomal rearrangement accompanied with transfection would not lead to growth advantages in the HLA-deficient T cells.

Although evaluation of HLA-deficient T cells’ efficacy in more clinically relevant settings needs to be preceded, our approach to target HLA expression on allogeneic cell surfaces could be applied more broadly to other therapeutic strategies, as exemplified in our data showing ex vivo stimulated NK cells with high HLA-II expression (Supplementary figure S2). Retinal pigment epithelial (RPE) cells are also explored to be used as allografts for the treatment of ocular diseases, and are reported to upregulate HLA-II in the presence of IFN-γ-mediated inflammatory responses^46^. Our engineering strategy may potentially be considered for the ablation of HLA molecules on those cells to control the host Th1 immune response^18^ for successful engraftment without HLA matching and/or conventional immune suppression^47–51^.

Our findings expand upon the increasingly recognized potential of allogeneic cell therapeutics in diverse clinical settings by identifying novel gRNA sequences ablating the surface expression of HLA, including highly polymorphic HLA-II molecules. The HLA-negative human primary T cells generated by delivery of the newly identified gRNAs maintained T cell functionality and activation phenotypes while elicited dramatically diminished alloreactive inflammatory reactions in responders. As our study reminds us of an essential conceptual aspect of HLA barriers in production of universal donor cells, it can ultimately be exploited to design new therapeutic strategies that decrease the risk of graft rejection and improve the clinical outcomes.

## Methods

### gRNA design and synthesis

gRNAs for each target gene were designed using the web-based tools CHOPCHOP^20^, E-CRISP^21^, and CRISPR-ERA^22^. Genomic sequences of the HLA-DRA allele 01:01 and HLA-DQA allele 01:01, known HLA type for Raji, and HLA-DPA allele 01:03 were used for gRNA design. Hundreds of candidate sequences were obtained from the web-based tools, and sixty gRNA sequences for each target gene (B2M, HLA-DRA, HLA-DQA and HLA-DPA) were pre-selected from the list according to the criteria: 1) Sequences which were obtained from multiple tools, 2) Sequences which exist in an exon, and 3) Sequences which have high rank in each tool. gRNAs were transcribed in vitro using a GeneArt Precision gRNA Synthesis Kit (Thermo Fisher Scientific, A29377) according to the manufacturer’s protocol. gRNA targeting sequences used in the study are listed in Supplementary Table S2.

### Raji cell culture and transfection

Raji cells were maintained in RPMI 1640 (Gibco, A10491-01) supplemented with 10% heat-inactivated FBS (Gibco, 16000–044), Antibiotic–Antimycotic (Gibco, 15240) and β-mercaptoethanol (Gibco, 21985–023). A total of 7.5 μg of Cas9 protein (Toolgen, TGEN_CP1; gRNA screening experiment in Supplementary figure S1, Clontech, 632640; all other experiments) and 7.5 μg of gRNA per reaction were mixed and incubated at room temperature for 10 min to form the Cas9/gRNA complex. For multiplex genome editing, 7.5 μg of gRNA in total were used. 4 × 10^5^ Raji cells were transfected with the Cas9/gRNA complex by 4D-Nucleofector X Unit (Lonza, AAF-1002X) using the SG Cell Line 4D-Nucleofector X Kit S (Lonza, V4XC-3032) with the program DS-104. Following transfection, the cells were cultured at 37 °C in 5% CO_2_ for 7 days, and the transfection efficiency was analyzed by flow cytometry. Mismatch-specific nuclease-mediated mutagenesis analysis and targeted deep sequencing were further performed.

### Preparation of human PBMCs

The study was approved by the institutional review board of the Mogam Institute for Biomedical Research (MG-2018–10-01) and conducted according to the Declaration of Helsinki. Human PBMCs were obtained from healthy volunteers by leukapheresis from the National Red Cross Blood Center (Suwon, South Korea). The informed consent form was signed by all subjects. PBMCs were isolated by centrifugation on a Ficoll density gradient and stored in liquid nitrogen.

### Human T cell transfection and expansion

Cryopreserved human PBMCs from healthy donors were thawed, and CD3^+^ cells were isolated using human CD3 MicroBeads (Miltenyi Biotec, 130–050-101). Upon T cell isolation, the CD3^+^ T cells were initially cultured in X-VIVO15 (Lonza, BE02-060Q) supplemented with Dynabeads Human T-Activator CD3/CD28 (Gibco, 111.31D), 200 IU/mL IL-2 (Novartis, 502519AF) and 5% human plasma (Valley Biomedical, HP1050) in a culture bag (NIPRO, 87–352). On day 1, 37.5 μg of Cas9 protein and 37.5 μg of gRNA per reaction were mixed and incubated at room temperature for 10 min to form Cas9/gRNA complex. For multiplex genome editing, 37.5 μg of gRNA in total were used. The CD3^+^ T cells were taken out from the culture bag and 4 × 10^6^ cells were transfected with Cas9/gRNA complex by 4D-Nucleofector X Unit (Lonza, AAF-1002X) using P3 Primary Cell 4D-Nucleofector X Kit L (Lonza, V4XP-3024) with the program DN-100. Following transfection, the cells were cultured at 37 °C in 5% CO_2_, and fresh culture medium, X-VIVO15 (Lonza, BE02-060Q) supplemented with 200 IU/mL IL-2 (Novartis, 502519AF) and 5% human plasma (Valley Biomedical, HP1050), was added every 2 to 3 days to reach a density of 1 × 10^6^ cells/mL. The cell number and viability were documented using the automated fluorescence cell counter (NanoEntek, ADAM-MC). The Dynabeads Human T-Activator CD3/CD28, which was provided in the culture media at the beginning of the ex vivo expansion was not replenished further.

### Human NK cell and B cell expansion

Cryopreserved human PBMCs from healthy donors were thawed and cultured in CellGro SCGM (CellGenix, 2001) supplemented with 10 ng/mL OKT3 (eBioscience, 16–0037-85), 500 IU/mL IL-2 (Novartis, 502519AF) and 5% human plasma (Valley Biomedical, HP1050) in a culture bag (NIPRO, 87–352). The cells were cultured at 37 °C in 5% CO_2_, and fresh culture medium was added every 2 to 3 days to reach a density of 1 × 10^6^ cells/mL. The expression of the HLA molecules on CD56^+^ NK cells and CD19^+^ B cells was analyzed by flow cytometry on days 0 and 12 post expansion.

### Flow cytometry and cell sorting

Flow cytometry analyses were performed on a LSR Fortessa (BD), and cell sorting was performed on a FACSAria II (BD). For HLA detection on immune cells, the following antibodies were used: Mouse anti-human V450 CD4 (BD, 560345), APC-Cy7 CD8a (Tonbo, 25–0088-T100), BV421 CD56 (BioLegend, 318328), FITC CD19 (eBioscience, 11–0199-42), BV510 HLA-ABC (BioLegend, 311436), PE-Cy7 HLA-DR (eBioscience, 25–9952-42), and Alx647 HLA-DQ (BD, 564806). To detect HLA-DP, cells were labeled with mouse anti-human HLA-DP (Abcam, AB20897), washed and stained with PE goat anti-mouse IgG (eBioscience, 12–4010-82). For T cell phenotyping, the following antibodies were used: Mouse anti-human PE CD69 (BD, 555531), PE-Cy7 CD44 (BD, 560533), BV421 CD25 (BD, 562443), PE-Cy7 PD-1 (BioLegend, 329918), and BV421 TIGIT (BD, 747844).

### Mismatch-specific nuclease-mediated mutagenesis analysis

Genomic mutation was determined by a Guide-it Mutation Detection Kit (Clontech, 631443) according to the manufacturer’s protocol. Briefly, targeted regions from the gene-disrupted cells were PCR-amplified, and the PCR products were denatured and re-annealed. Then, the hybridized PCR products were digested with Guide-it Resolvase for 60 min at 37 °C and analyzed by agarose-gel electrophoresis. The primers used for PCR are listed in Supplementary Table S2.

### Targeted deep sequencing and data analysis

Targeted deep sequencing data were provided by ToolGen Indel analysis service. Off-target sites were identified using the CCTop CRISPR/Cas9 target online predictor^52^ and are listed in Supplementary Table S3. The target sites were amplified using Phusion High-Fidelity DNA Polymerase (NEB, M0530L) and specific primers (Supplementary Table S3) from purified genomic DNA. PCR amplicons were denatured with NaOH and subjected to paired-end sequencing using an Illumina MiSeq sequencer. Indel frequencies were calculated using the Cas-Analyzer.

### In vitro cytotoxicity analysis

On day 14 post expansion, 1 × 10^6^ engineered T cells were stimulated with Cell Activation Cocktail (without Brefeldin A) (BioLegend, 423301) or Dynabeads Human T-Activator CD3/CD28 in 96-well round-bottom plates for 5 h at 37 °C. After 1 h of incubation, a mixture of GolgiStop (BD, 554724) and GolgiPlug (BD, 555029) was added. To detect T cell degranulation, mouse anti-human PE-Cy7 CD107a antibody (BD, 561348) or PE-Cy7 isotype (Mouse IgG1κ, BD, 557872) (1 µL/well) were treated during the culture. The cells were then stained with mouse anti-human FITC CD3 (BD, 561807), APC-Cy7 HLA-ABC (BioLegend, 311426), PE HLA-DRDPDQ (Miltenyi Biotec, 130–104-827) and LIVE/DEAD Fixable Aqua Dead Cell Stain Kit (Invitrogen, L34957) for dead cell exclusion. Using a Fixation/Permeabilization Solution Kit (BD, 554714), the cells were intracellularly stained with BV421 IFN-γ (BD, 562988) and Alx647 TNF-α (BioLegend, 502916) antibodies and analyzed by flow cytometry.

### In vitro allogeneic mixed lymphocyte reaction (MLR) and overnight intracellular staining (ICS) assay

On day 14 post expansion, the B2M gRNA treated T cells, quadruple-gene-edited T cells and non-targeting gRNA treated T cells were stained with anti-human PE HLA-ABC (Miltenyi Biotec, 130–120-055) and APC HLA-DRDPDQ (Miltenyi Biotec, 130–104-824) and were sorted based on their HLA-I and HLA-II expression using a FACSAria II. The sorted cells were incubated in the 37 °C incubator overnight for resting and subjected to γ-irradiation (30 Gy) before the MLR assays to minimize the alloreactive responses. Another set of cryopreserved allogeneic human PBMCs was thawed on the day of MLR and stained with CellTrace Violet Cell Proliferation Kit (Thermo Fisher, C34557). To measure the alloreactive responses of responder PBMCs against engineered T cells, we performed in vitro MLR assays where 2.5 × 10^4^ CTV-labeled whole allogeneic PBMCs were co-cultured with the 2.5 × 10^4^ expanded, sorted T cells in 96-well round-bottom plates. A mixture of GolgiStop (BD, 554724) and GolgiPlug (BD, 555029) was added into the MLR wells. After a 12-h incubation, the cells were stained with mouse anti-human APC-Cy7 CD3 (BioLegend, 300426) and 7-AAD (BD, 559925). Using a Fixation/Permeabilization Solution Kit (BD, 554714), the cells were intracellularly stained with PE IFN-γ (BD, 554701) and PE-Cy7 TNF-α (eBioscience, 25–7349-82), and intracellular TNF-α and IFN-γ production within the CTV^+^ CD3^+^ were analyzed by flow cytometry.

### Detection of chromosomal rearrangements by end-point PCR

On day 14 post expansion, HLA-I/II-negative cells were sorted from quadruple-gene-edited T cells using a FACSAria II (BD Biosciences). Sorted HLA-I/II-negative cells were cultured at 37 °C in 5% CO_2_, and fresh culture medium, X-VIVO15 (Lonza, BE02-060Q) supplemented with 200 IU/mL IL-2 (Novartis, 502519AF) and 5% human plasma (Valley Biomedical, HP1050), was added every 2 to 3 days to reach a density of 1 × 10^6^ cells/mL. Genomic DNA was extracted from untransfected T cells and HLA-I/II-negative T cells on day 0, 7 and 14 after sorting with the PureLink Genomic DNA Mini Kit (Invitrogen, K182002) according to the manufacturer’s instructions. PCRs were performed using the Phusion High-Fidelity DNA Polymerase (NEB, M0530L) according to the manufacturer’s instructions with 20 ng of DNA per reaction with the following cycling conditions: one cycle of 98 °C for 30 s, 30 cycles of 98 °C for 10 s, 62 °C for 30 s, 72 °C for 30 s, and one cycle of 72 °C for 10 min. The primers used for PCR are listed in Supplementary Table S4. All 20 possible primer combinations B2M:DRA, B2M:DQA1, B2M:DQA2, B2M:DPA, DQA:B2M and so forth, were tested to detect rearrangement. HPRT was used as an endogenous control. Synthetic double-stranded DNA containing tandem sequences for the 5 forward primers and for the 5 reverse primers were inserted into pUC19 plasmid and used as a positive control. Samples were loaded on 1.5% agarose gel.

## Supplementary information

Supplementary Information 1.
