# Supplementary material for: Abrogation of HLA surface expression using CRISPR/Cas9 genome editing: a step toward universal T cell therapy
Source: Sci Rep. 2020 Oct 20;10:17753. doi: 10.1038/s41598-020-74772-9 (PMC7576162; doi:10.1038/s41598-020-74772-9)
Supplement: Supplementary file 1 — Supplementary Information 1. [file 41598_2020_74772_MOESM1_ESM.docx]

**Supplementary Information**

**Abrogation of HLA surface expression using CRISPR/Cas9 genome editing: a step toward universal T cell therapy**

Jeewon Lee^$^, Joong Hyuk Sheen^$^, Okjae Lim, Yunjung Lee, Jihye Ryu, Duckhyang Shin, Yu Young Kim, Munkyung Kim^*^

MOGAM Institute for Biomedical Research, 93, 30beon-gil, Ihyeon-ro, Giheung-gu, Yongin-si, Gyeonggi-do, South Korea

^$^These authors contributed equally to this work.

*Corresponding author:

Munkyung Kim

MOGAM Institute for Biomedical Research

93, 30beon-gil, Ihyeon-ro, Giheung-gu, Yongin-si, Gyeonggi-do

16924, South Korea

Tel. +82 31 260 0825

Fax. +82 31 260 9870

E-mail: munkyung.kim@mogam.re.kr

**
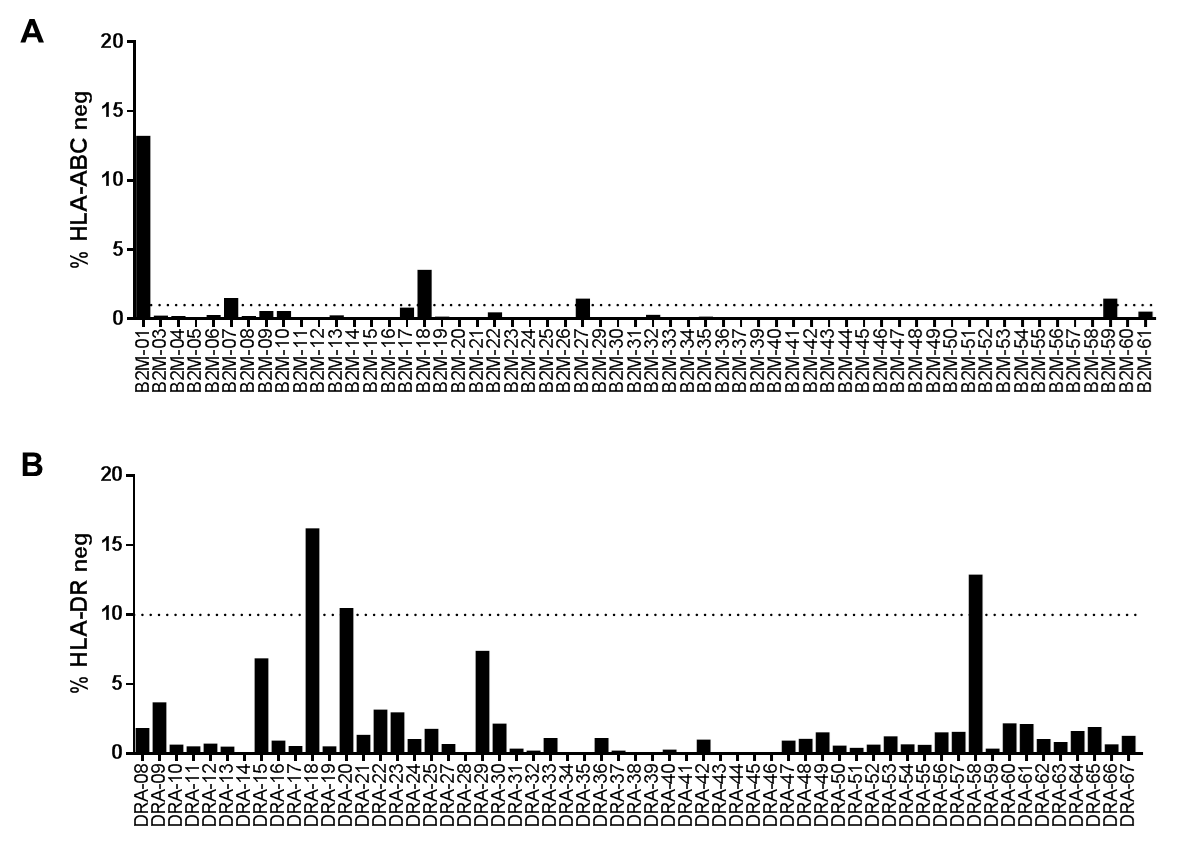
**

**
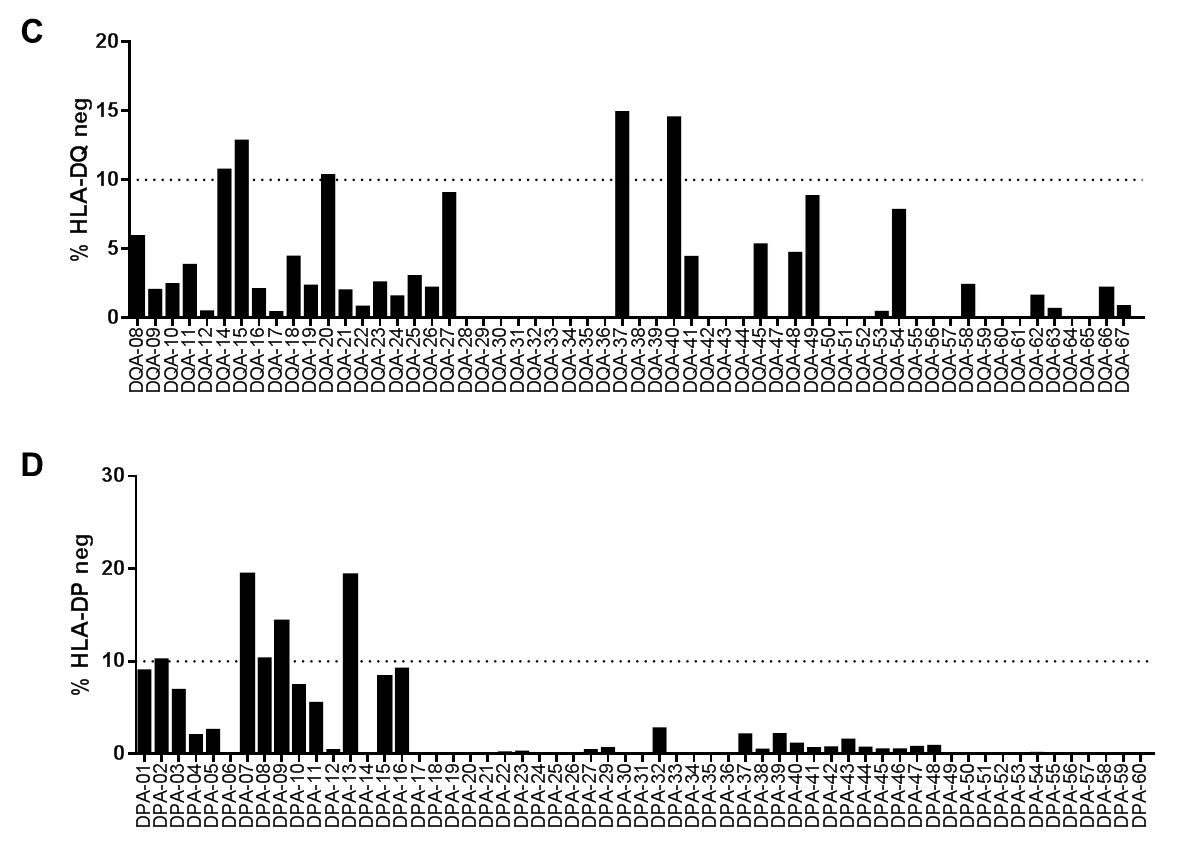
**

**Supplementary figure S1.** **Target deletion efficiency of designed gRNAs for each target gene.** Quantification of flow cytometry analyses of HLA expression on Raji cells transfected with pre-selected gRNAs targeting B2M (**A**), HLA-DRA (**B**), HLA-DQA (**C**) or HLA-DPA (**D**) together with Cas9 protein. Horizontal dot lines indicate the internal criteria of deletion efficiency for further selection.

**
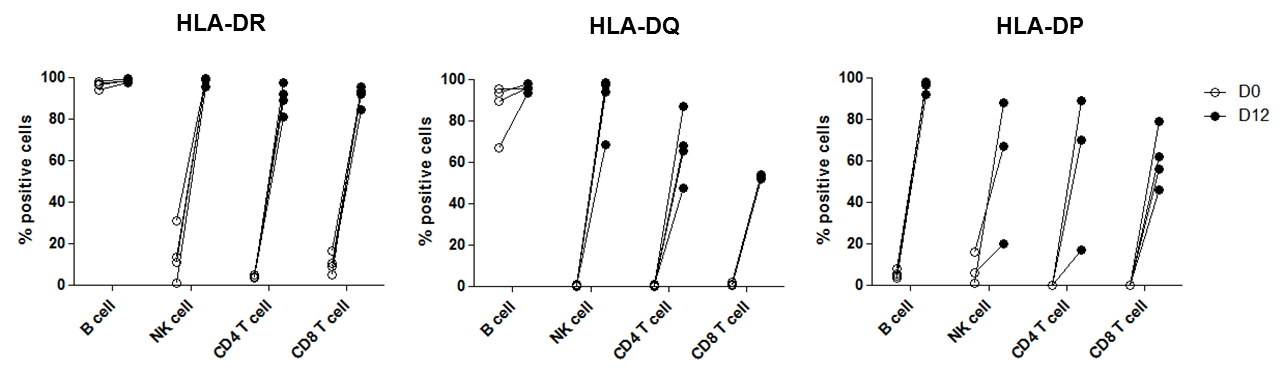
**

**Supplementary figure S2. Increased surface expression of HLA-II in immune cell subsets pre-/post-expansion.** Quantification of flow cytometry analyses of HLA-II expression in B, NK and T cells before and after 12 days of *ex vivo* expansion. Pooled results from three independently performed experiments, total n=3 or 4 different donors.


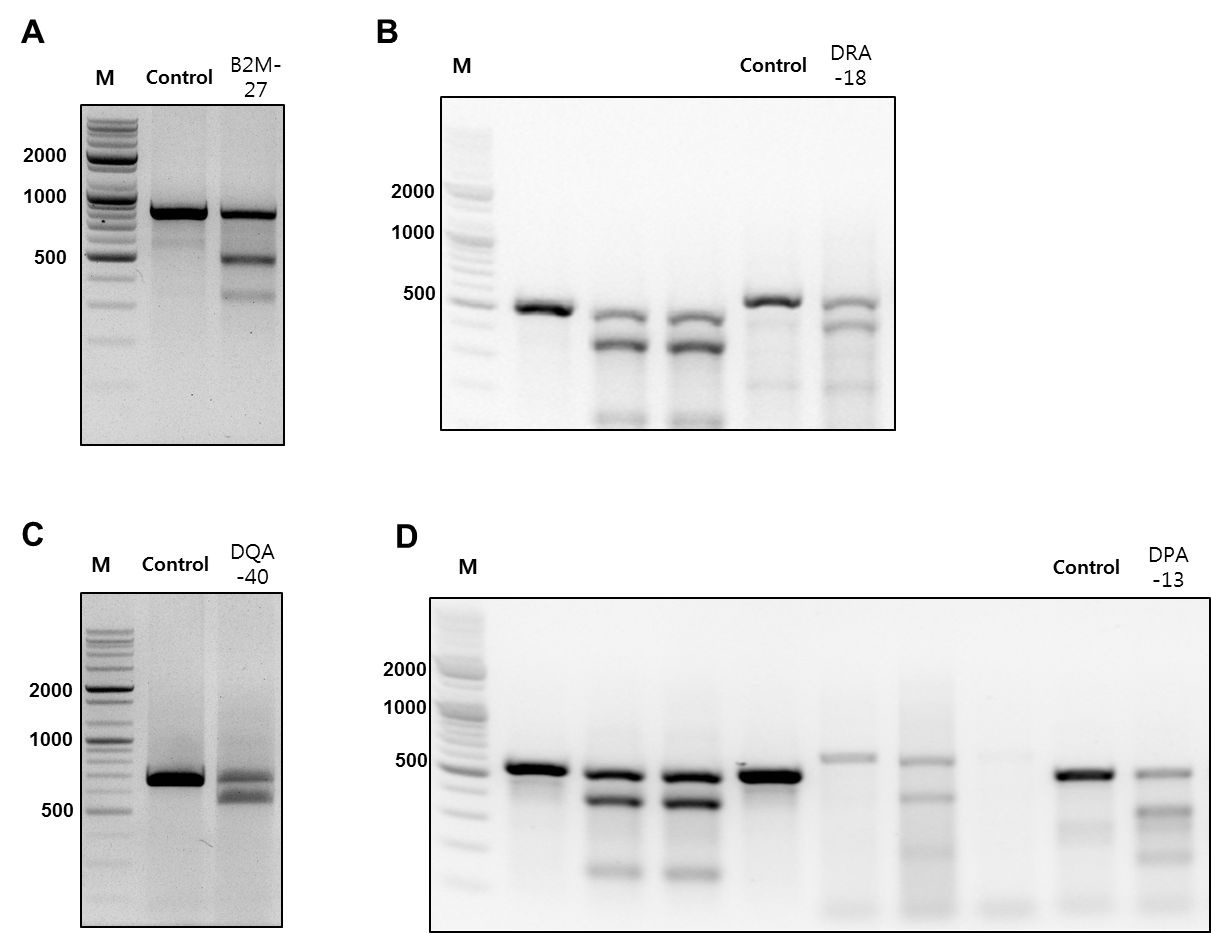


**Supplementary figure S3.** Display of full-length gel images of cropped images presented in figure 1B. Full length gel associated to B2M-27 gRNA (**A**), DRA-18 gRNA (**B**), DQA-40 gRNA (**C**) or DPA-13 gRNA (**D**) is shown. M, DNA Marker

**Supplementary figure S4. Fold expansion of T cells with or without HLA engineering.** Cell numbers of untransfected, non-targeting gRNA/Cas9-transfected and quadruple-HLA- gRNAs/Cas9-transfected CD3^+^ T cells were measured every 2 to 3 days during *ex vivo* expansion. Relative fold expansion was calculated as cell numbers at indicated time points with respect to the cell numbers on expansion day 1 (day of Cas9/gRNA transfection). Pooled results from three independently performed experiments are shown, total n=6 different donors. Error bars indicate mean+SEM.


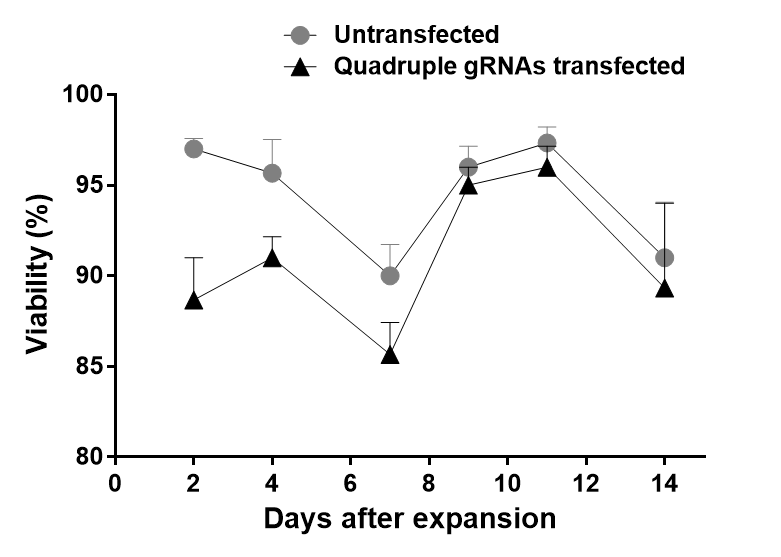


**Supplementary figure S5. Viability of primary T cells with or without HLA engineering during *ex vivo* expansion.** Viability of untransfected and quadruple gRNAs/Cas9-transfected T cells was assessed every 2 to 3 days during *ex vivo* expansion culture using the automated fluorescence cell counter. Pooled results from 3 different PBMC donors. Error bars indicate mean+SEM.


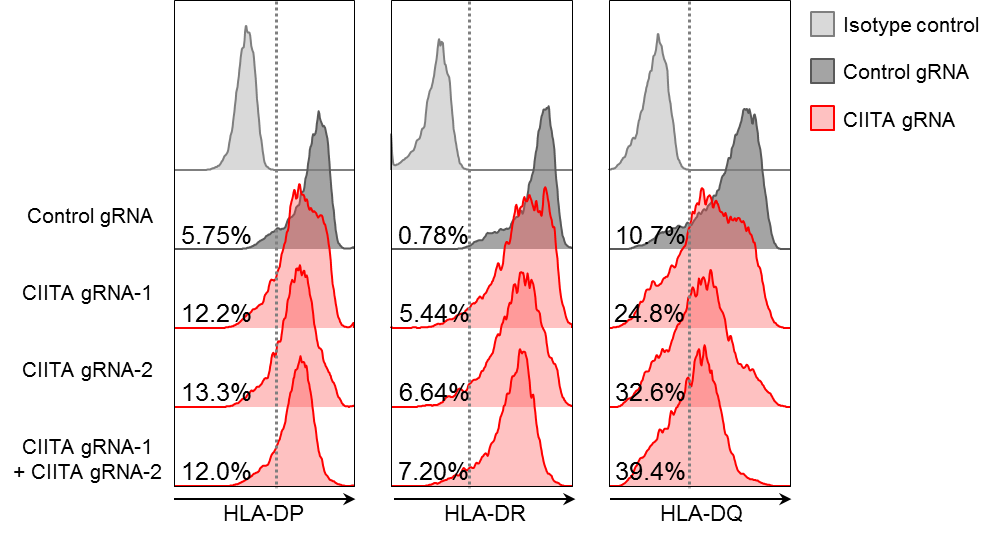


**Supplementary figure S6. HLA-II ablation by CIITA-targeting gRNAs in Raji cells.** Flow cytometry analyses of HLA-II (HLA-DP, HLA-DR and HLA-DQ) expression in Raji cells 7 days after indicated gRNA/Cas9 transfection. A representative histogram image from two independent experiments is shown. A number in each plot indicates % of HLA-II negative cells out of total cells. HLA-II staining was determined by the isotype control staining (light grey).

**
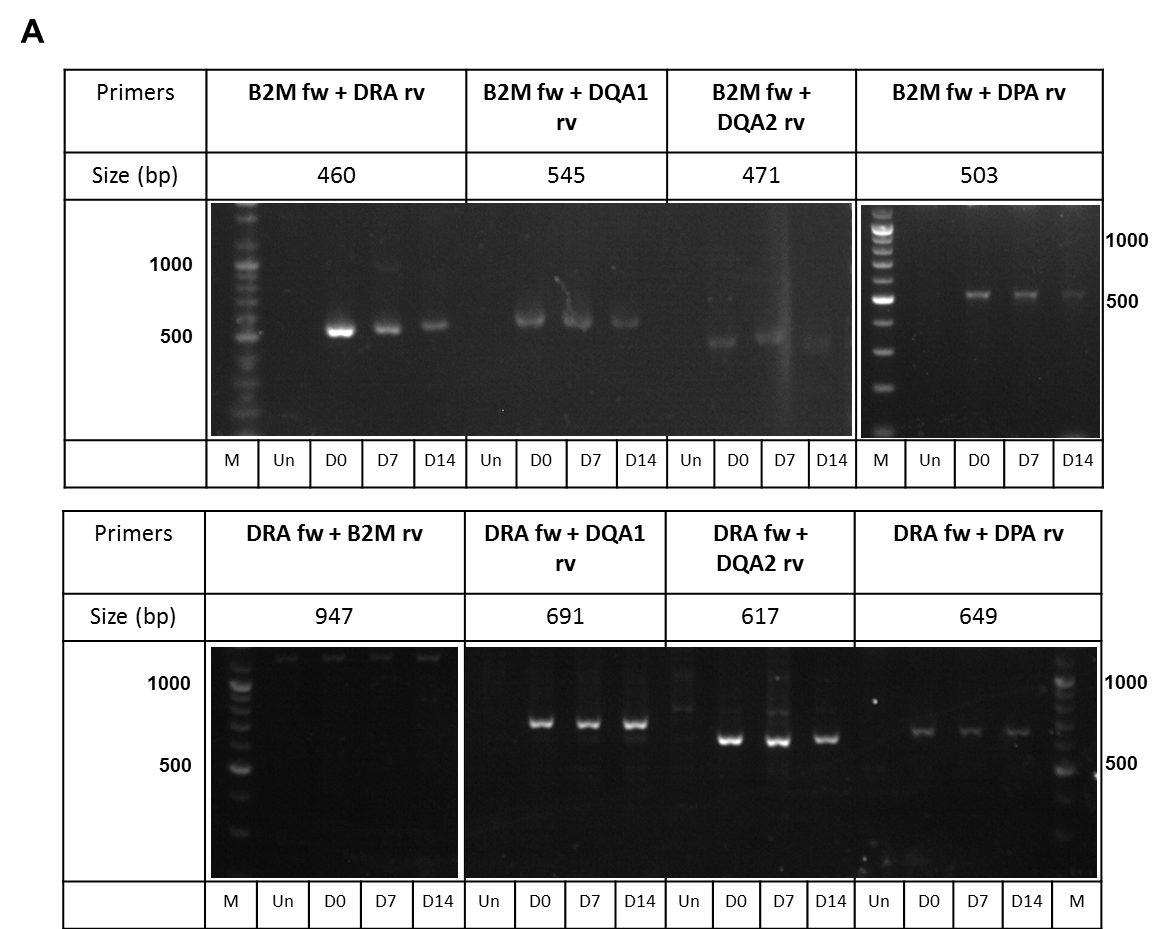
**

**
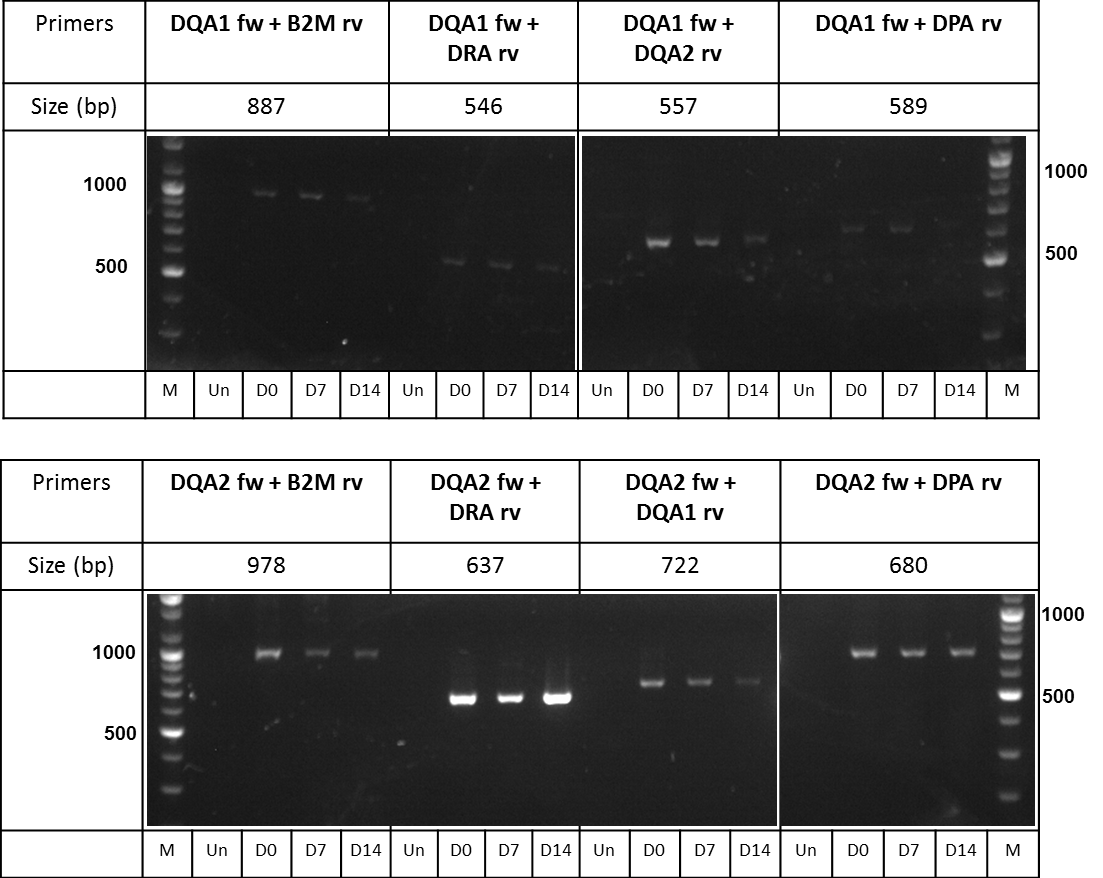
**

**
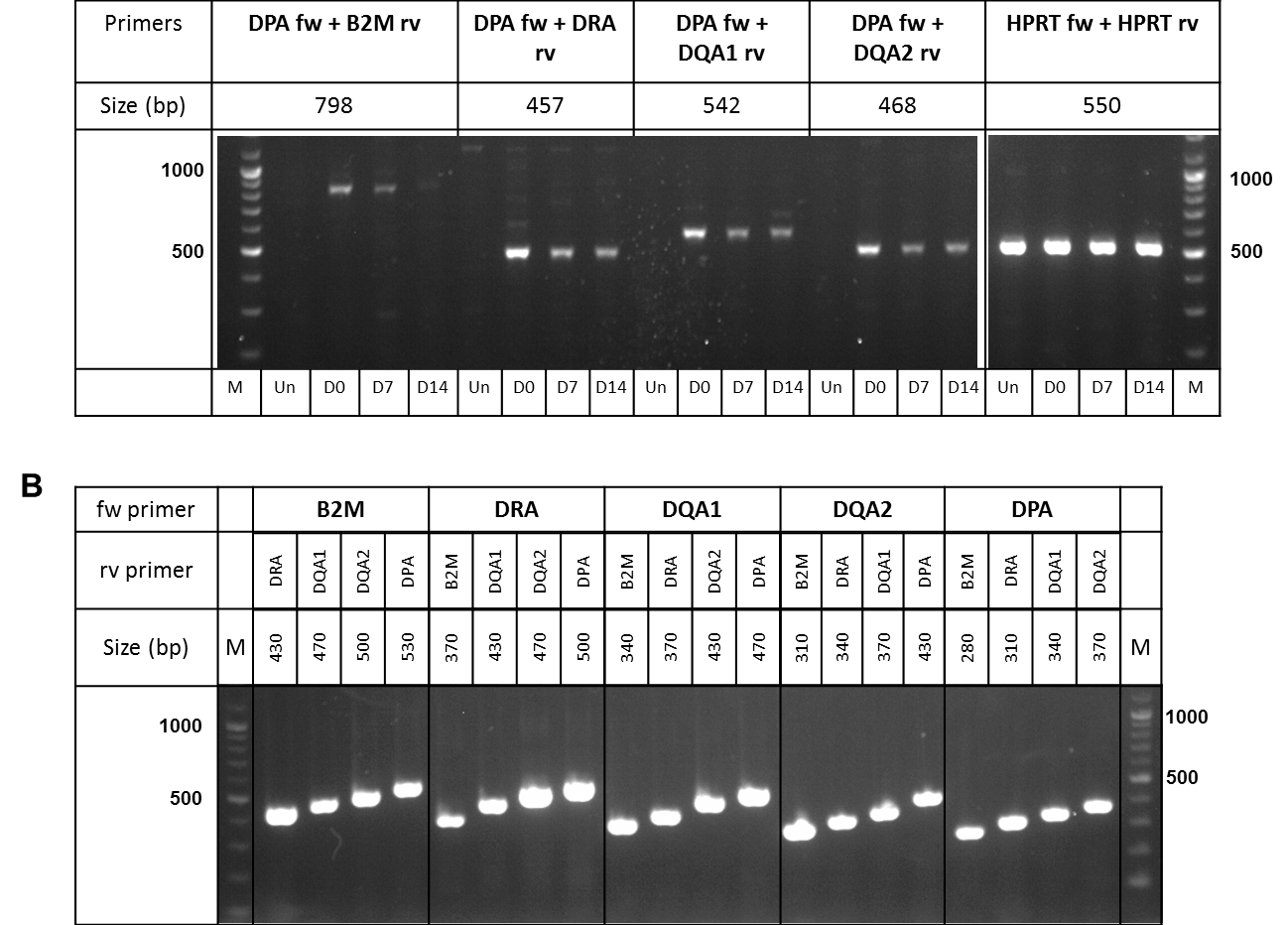
**

**
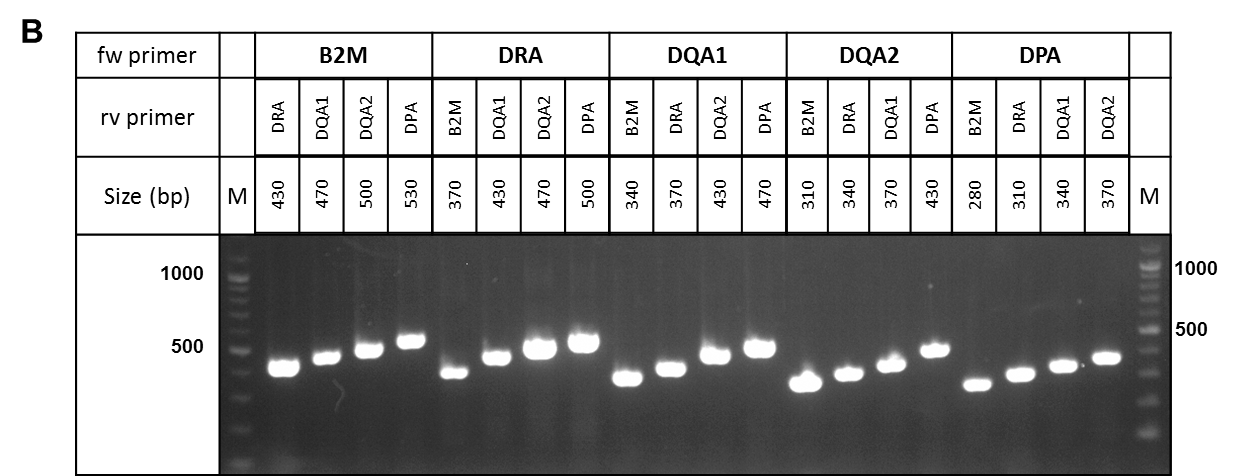
**

**Supplementary figure S7. Detection of chromosomal rearrangements in quadruple-gene-edited T cells. A,** Agarose gel electrophoresis of PCR-amplified products from genomic DNA extracted from untransfected (Un) or HLA-I/II-negative T cells (D0, D7, D14). HLA-I/II-negative T cells were sorted on day 14 post expansion from quadruple-HLA gRNAs/Cas9 transfected T cells and genomic DNA was extracted on day 0, 7 and 14 after sorting (D0, D7, D14). Amplicons were obtained using indicated primer sets. All rearrangements tested were detected except one translocation (DRA:B2M). Representative data from 3 different PBMC donors is shown. **B,** Agarose gel electrophoresis of control PCR with positive control plasmid. Amplicons were obtained using indicated primer sets. fw, forward; rv, reverse; M, DNA Marker.

**Supplementary Table S1. The numbers of HLA-II α chain gene alleles and coverage of selected gRNAs.** Allele numbers from the IMGT/HLA database, assigned as of Sep 2019.

|  | **DRA** | **DQA1** | **DPA1** |
| --- | --- | --- | --- |
| **HLA alleles numbers** | 29 | 216 | 161 |
| **Number of alleles whose genomic sequence is available** | 28 | 140 | 86 |
| **Number of alleles matched with gRNA sequence** | DRA-18 (28/28) DRA-20 (28/28) DRA-58 (28/28) | DQA-14 (104/140) DQA-15 (104/140) DQA-37 (114/140) DQA-40 (140/140) | DPA-07 (80/86) DPA-13 (86/86) |

**Supplementary Table S2. gRNA sequences and PCR primer sequences for genomic mutation detection.**

| **Target** | **gRNA sequences** | **PCR primer forward** | **PCR primer reverse** |
| --- | --- | --- | --- |
| **B2M** | | | |
| B2M-01 | GAGTAGCGCGAGCACAGCTA |  |  |
| B2M-03 | CTCGCGCTACTCTCTCTTTC |  |  |
| B2M-04 | GCATACTCATCTTTTTCAGT |  |  |
| B2M-05 | GCTACTCTCTCTTTCTGGCC |  |  |
| B2M-06 | GGCATACTCATCTTTTTCAG |  |  |
| B2M-07 | GGCCACGGAGCGAGACATCT |  |  |
| B2M-08 | GGCCGAGATGTCTCGCTCCG |  |  |
| B2M-09 | TCACGTCATCCAGCAGAGAA |  |  |
| B2M-10 | ACAAAGTCACATGGTTCACA |  |  |
| B2M-11 | AGTCACATGGTTCACACGGC |  |  |
| B2M-12 | AAGTCAACTTCAATGTCGGA |  |  |
| B2M-13 | CATACTCATCTTTTTCAGTG |  |  |
| B2M-14 | TCCTGAATTGCTATGTGTCT |  |  |
| B2M-15 | CGTGAGTAAACCTGAATCTT |  |  |
| B2M-16 | TTGGAGTACCTGAGGAATAT |  |  |
| B2M-17 | AGGGTAGGAGAGACTCACGC |  |  |
| B2M-18 | ACAGCCCAAGATAGTTAAGT |  |  |
| B2M-19 | ATACTCATCTTTTTCAGTGG |  |  |
| B2M-20 | TGGAGTACCTGAGGAATATC |  |  |
| B2M-21 | AAGAAAAGGAAACTGAAAAC |  |  |
| B2M-22 | AAGAAGGCATGCACTAGACT |  |  |
| B2M-23 | ACATGTAAGCAGCATCATGG |  |  |
| B2M-24 | ACCCAGACACATAGCAATTC |  |  |
| B2M-25 | ACTTGTCTTTCAGCAAGGAC |  |  |
| B2M-26 | CAAGCCAGCGACGCAGTGCC |  |  |
| B2M-27 | CACAGCCCAAGATAGTTAAG | CCAAGTTAGCCCCAAGTGAA | ACCTACTGGCTTCCTCTAGCT |
| B2M-29 | CATCACGAGACTCTAAGAAA |  |  |
| B2M-30 | CGCAGTGCCAGGTTAGAGAG |  |  |
| B2M-31 | CTAACCTGGCACTGCGTCGC |  |  |
| B2M-32 | GAAAGTCCCTCTCTCTAACC |  |  |
| B2M-33 | GAGACATGTAAGCAGCATCA |  |  |
| B2M-34 | GAGTCTCGTGATGTTTAAGA |  |  |
| B2M-35 | GCAGTGCCAGGTTAGAGAGA |  |  |
| B2M-36 | TAAGAAGGCATGCACTAGAC |  |  |
| B2M-37 | TCGATCTATGAAAAAGACAG |  |  |
| B2M-39 | TTCAGACTTGTCTTTCAGCA |  |  |
| B2M-40 | TTCCTGAATTGCTATGTGTC |  |  |
| B2M-41 | TAAGAAAAGGAAACTGAAAA |  |  |
| B2M-42 | CTGGCACTGCGTCGCTGGCT |  |  |
| B2M-43 | TGCGTCGCTGGCTTGGAGAC |  |  |
| B2M-44 | GCTGGCTTGGAGACAGGTGA |  |  |
| B2M-45 | AGACAGGTGACGGTCCCTGC |  |  |
| B2M-46 | CAATCAGGACAAGGCCCGCA |  |  |
| B2M-47 | CCTGCGGGCCTTGTCCTGAT |  |  |
| B2M-48 | CCAATCAGGACAAGGCCCGC |  |  |
| B2M-49 | CGGGCCTTGTCCTGATTGGC |  |  |
| B2M-50 | GGGCCTTGTCCTGATTGGCT |  |  |
| B2M-51 | GTGCCCAGCCAATCAGGACA |  |  |
| B2M-52 | AAACGCGTGCCCAGCCAATC |  |  |
| B2M-53 | GGGCACGCGTTTAATATAAG |  |  |
| B2M-54 | CACGCGTTTAATATAAGTGG |  |  |
| B2M-55 | TATAAGTGGAGGCGTCGCGC |  |  |
| B2M-56 | AAGTGGAGGCGTCGCGCTGG |  |  |
| B2M-57 | AGTGGAGGCGTCGCGCTGGC |  |  |
| B2M-58 | TTCCTGAAGCTGACAGCATT |  |  |
| B2M-59 | TCCTGAAGCTGACAGCATTC |  |  |
| B2M-60 | TGGGCTGTGACAAAGTCACA |  |  |
| B2M-61 | ACTCTCTCTTTCTGGCCTGG |  |  |
| **DRA** | | | |
| DRA-08 | AAGAAGAAAATGGCCATAAG |  |  |
| DRA-09 | AATCATGGGCTATCAAAGGT |  |  |
| DRA-10 | AGCTGTGCTGATGAGCGCTC |  |  |
| DRA-11 | ATAAGTGGAGTCCCTGTGCT |  |  |
| DRA-12 | ACTTATGGCCATTTTCTTCT |  |  |
| DRA-13 | ATGATGAAAAATCCTAGCAC |  |  |
| DRA-14 | CAGAGCGCCCAAGAAGAAAA |  |  |
| DRA-15 | CAGGAATCATGGGCTATCAA |  |  |
| DRA-16 | CTTATGGCCATTTTCTTCTT |  |  |
| DRA-17 | GACTGTCTCTGACACTCCTG |  |  |
| DRA-18 | GAGCCTCTTCTCAAGCACTG | AATTTCTTGGGGAGGGGGTG | AGCTGGATAGTAGGAGAAGACAGT |
| DRA-19 | GATAGTGGAACTTGCGGAAA |  |  |
| DRA-20 | GATGAGCGCTCAGGAATCAT |  |  |
| DRA-21 | GCTATCAAAGGTAGGTGCTG |  |  |
| DRA-22 | GTTACCTCTGGAGGTACTGG |  |  |
| DRA-23 | TAGCACAGGGACTCCACTTA |  |  |
| DRA-24 | TGATGAAAAATCCTAGCACA |  |  |
| DRA-25 | TGATGAGCGCTCAGGAATCA |  |  |
| DRA-27 | TTTGCCAGCTTTGAGGCTCA |  |  |
| DRA-28 | AACTATACTCCGATCACCAA |  |  |
| DRA-29 | AGAAGAACATGTGATCATCC |  |  |
| DRA-30 | AGCAGAGAGGGAGGTACCAT |  |  |
| DRA-31 | AGCGCTTTGTCATGATTTCC |  |  |
| DRA-32 | AGCTGTGGACAAAGCCAACC |  |  |
| DRA-33 | AGGGAGGTACCATTGGTGAT |  |  |
| DRA-34 | ATAAACTCGCCTGATTGGTC |  |  |
| DRA-35 | ATTGGTGATCGGAGTATAGT |  |  |
| DRA-36 | CCATGTGGATATGGCAAAGA |  |  |
| DRA-37 | CTTTGAGGCTCAAGGTGCAT |  |  |
| DRA-38 | CTTTGTCATGATTTCCAGGT |  |  |
| DRA-39 | GGATATGGCAAAGAAGGAGA |  |  |
| DRA-40 | TATCTGAATCCTGACCAATC |  |  |
| DRA-41 | TGAGATTTTCCATGTGGATA |  |  |
| DRA-42 | TGATCACATGTTCTTCTGAA |  |  |
| DRA-43 | TGCACCTTGAGCCTCAAAGC |  |  |
| DRA-44 | TGCATTGGCCAACATAGCTG |  |  |
| DRA-45 | TGGACGATTTGCCAGCTTTG |  |  |
| DRA-46 | TGGCAAAGAAGGAGACGGTC |  |  |
| DRA-47 | TGGTGATGAGATTTTCCATG |  |  |
| DRA-48 | AATGTCACGTGGCTTCGAAA |  |  |
| DRA-49 | AGACAAGTTCACCCCACCAG |  |  |
| DRA-50 | CAATCCCTTGATGATGAAGA |  |  |
| DRA-51 | GAACGCAGGGGGCCTCTGTA |  |  |
| DRA-52 | CTGAGGACGTTTACGACTGC |  |  |
| DRA-53 | GCGGAAAAGGTGGTCTTCCC |  |  |
| DRA-54 | GGACGTTTACGACTGCAGGG |  |  |
| DRA-55 | GTCGTAAACGTCCTCAGTTG |  |  |
| DRA-56 | GTGAGCACAGTTACCTCTGG |  |  |
| DRA-57 | GTGTCCCCCAGTACCTCCAG |  |  |
| DRA-58 | TGAGGACGTTTACGACTGCA |  |  |
| DRA-59 | AATGGAAAACCTGTCACCAC |  |  |
| DRA-60 | AGTGGAACTTGCGGAAAAGG |  |  |
| DRA-61 | ATGAAACAGATGAGGACGTT |  |  |
| DRA-62 | CAGAGACAGTCTTCCTGCCC |  |  |
| DRA-63 | CGTGACATTGACCACTGGTG |  |  |
| DRA-64 | TATGAAACAGATGAGGACGT |  |  |
| DRA-65 | TCTGACACTCCTGTGGTGAC |  |  |
| DRA-66 | AAACGTCCTCAGTTGAGGGC |  |  |
| DRA-67 | TCGTAAACGTCCTCAGTTGA |  |  |
| **DQA** | | | |
| DQA-08 | TTAGGATCATCCTCTTCCCA |  |  |
| DQA-09 | AACTCTACCGCTGCTACCAA |  |  |
| DQA-10 | ACAATGTCTTCACCTCCACA |  |  |
| DQA-11 | ACCACCGTGATGAGCCCCTG |  |  |
| DQA-12 | ACCCAGTGTCACGGGAGACT |  |  |
| DQA-14 | ACCTCCACAGGGGCTCATCA |  |  |
| DQA-15 | CAATGTCTTCACCTCCACAG |  |  |
| DQA-16 | CACAATGTCTTCACCTCCAC |  |  |
| DQA-17 | CAGTACACCCATGAATTTGA |  |  |
| DQA-18 | CTCTGTGAGCTCTGACATAG |  |  |
| DQA-19 | CTGTGGAGGTGAAGACATTG |  |  |
| DQA-20 | GGCTGGAATCTCAGGCTCTG |  |  |
| DQA-21 | GTTGGGCTGACCCAGTGTCA |  |  |
| DQA-22 | TCATGGGTGTACTGGCCAGA |  |  |
| DQA-23 | TCCAAGTCTCCCGTGACACT |  |  |
| DQA-24 | TCCACAGGGGCTCATCACGG |  |  |
| DQA-25 | TGTGGAGGTGAAGACATTGT |  |  |
| DQA-26 | TTCCAAGTCTCCCGTGACAC |  |  |
| DQA-27 | TTGGGCTGACCCAGTGTCAC |  |  |
| DQA-28 | AACATCACATGGCTGAGCAA |  |  |
| DQA-29 | ACATCACATGGCTGAGCAAT |  |  |
| DQA-30 | AGCCATGTGATGTTGACCAC |  |  |
| DQA-31 | AGGAATGATCACTCTTGGAG |  |  |
| DQA-32 | ATCACTCTTGGAGAGGAAGC |  |  |
| DQA-33 | ATGACTGCAAGGTGGAGCAC |  |  |
| DQA-34 | CAAGGTGGAGCACTGGGGCC |  |  |
| DQA-35 | CATCAAATTCATGGGTGTAC |  |  |
| DQA-36 | CATGTGATGTTGACCACAGG |  |  |
| DQA-37 | CCTCACCACAGAGGTTCCTG |  |  |
| DQA-38 | CTCATCTCCATCAAATTCAT |  |  |
| DQA-39 | CTCCTGTGGTCAACATCACA |  |  |
| DQA-40 | GAAGAAGGAATGATCACTCT | TCCCTCCATACCAGGGTTCA | AACTCATCCTTACCCCAGTGT |
| DQA-41 | GACTGCAAGGTGGAGCACTG |  |  |
| DQA-42 | GAGGTAACTGATCTTGAAGA |  |  |
| DQA-43 | GGACAACATCTTTCCTCCTG |  |  |
| DQA-44 | GTGCTGTTTCCTCACCACAG |  |  |
| DQA-45 | TCTTCTGAAACACTGGGGTA |  |  |
| DQA-47 | TTCATGGGTGTACTGGCCAG |  |  |
| DQA-48 | AGAGACTGTGGTCTGCGCCC |  |  |
| DQA-49 | GACATAGGGGCTGGAATCTC |  |  |
| DQA-50 | GAGACTGTGGTCTGCGCCCT |  |  |
| DQA-51 | GGCCTCGTGGGCATTGTGGT |  |  |
| DQA-52 | GGGCCTCGTGGGCATTGTGG |  |  |
| DQA-53 | GTCAGAGCTCACAGAGACTG |  |  |
| DQA-54 | GTCTCTGTGAGCTCTGACAT |  |  |
| DQA-55 | GTGAGCTCTGACATAGGGGC |  |  |
| DQA-56 | GTTGGTGCTTCCAGACACCA |  |  |
| DQA-57 | TCTCTGTGAGCTCTGACATA |  |  |
| DQA-58 | TGACTGCAAGGTGGAGCACT |  |  |
| DQA-59 | TGCCCACCACAATGCCCACG |  |  |
| DQA-60 | TGGAAGCACCAACTGAACGC |  |  |
| DQA-61 | TGTGGGCCTCGTGGGCATTG |  |  |
| DQA-62 | TTACCCCAGTGTTTCAGAAG |  |  |
| DQA-63 | TTGGAAAACACTGTGACCTC |  |  |
| DQA-64 | TTGGTGCTTCCAGACACCAA |  |  |
| DQA-65 | AAACAAAGCTCTGCTGCTGG |  |  |
| DQA-66 | AAATCTCATCAGCAGAAGGG |  |  |
| DQA-67 | CTAAACAAAGCTCTGCTGCT |  |  |
| DQA-15 | CAATGTCTTCACCTCCACAG |  |  |
| **DPA** | | | |
| DPA-01 | TCTATGCGTCTGTACAAACG |  |  |
| DPA-02 | GTACAGACGCATAGACCAAC |  |  |
| DPA-03 | TACAGACGCATAGACCAACA |  |  |
| DPA-04 | GAAGGAGACCGTCTGGCATC |  |  |
| DPA-05 | GGAGACCGTCTGGCATCTGG |  |  |
| DPA-06 | GTCTGGCATCTGGAGGAGTT |  |  |
| DPA-07 | GTGGTTGGAACGCTGGATCA |  |  |
| DPA-08 | GTCTTCAGGGCGCATGTTGT |  |  |
| DPA-09 | TGTCTTCAGGGCGCATGTTG |  |  |
| DPA-10 | TCTTCAGGGCGCATGTTGTG |  |  |
| DPA-11 | GTTGCATACCCCAGTGCTTG |  |  |
| DPA-12 | GACCTTTGTGCCCTCAGCAG |  |  |
| DPA-13 | GAGACTCAGCAGGAAAGCCA | TGTGAACTGGAGCTCTCTTGA | TATGAGGGCCAGAGGGAACAT |
| DPA-14 | GAGCCTCAAAGGAAAAGGCT |  |  |
| DPA-15 | GATCTTGAGAGCCCTCTCCT |  |  |
| DPA-16 | GCCATCAAGGGTGAGTGCTC |  |  |
| DPA-17 | GCCATGACCCCCGGGCCCAG |  |  |
| DPA-18 | GCCCAGCTCCACAGGCTCCT |  |  |
| DPA-19 | GCCCTGAGCCTCAAAGGAAA |  |  |
| DPA-20 | GCCTTTTCCTTTGAGGCTCA |  |  |
| DPA-21 | GCGTTCTGGCCATGACCCCC |  |  |
| DPA-22 | GCTTTCCTGCTGAGTCTCCG |  |  |
| DPA-23 | GGAAACACGGTCACCTCAGG |  |  |
| DPA-24 | GGACTTCTATGACTGCAGGG |  |  |
| DPA-25 | GGAGACTGTGCTCTGTGCCC |  |  |
| DPA-26 | GGCCATGACCCCCGGGCCCA |  |  |
| DPA-27 | GGCCTAGTCGGCATCATCGT |  |  |
| DPA-29 | GGGAAACACGGTCACCTCAG |  |  |
| DPA-30 | GGGCCTAGTCGGCATCATCG |  |  |
| DPA-31 | GTCATAGAAGTCCTCTGCTG |  |  |
| DPA-32 | GTCCTCTGCTGAGGGCACAA |  |  |
| DPA-33 | GTGGAAGCTGTAATCTGTTC |  |  |
| DPA-34 | GTGGGAAGAACTTGTCAATG |  |  |
| DPA-35 | GTTGGTGGCCTGAGTGTGGT |  |  |
| DPA-36 | GTTGTCTCAGGCATCTGGAT |  |  |
| DPA-37 | GCTGAGTCTCCGAGGAGCTG |  |  |
| DPA-38 | TCTCTACTGTCTTTATGCAG |  |  |
| DPA-39 | TATGGAACATTCTGTCTTCA |  |  |
| DPA-40 | TCAAGATCACAGCTCTGATA |  |  |
| DPA-41 | TCAAACATAAACTCCCCTGT |  |  |
| DPA-42 | TACCGTTGGTGGCCTGAGTG |  |  |
| DPA-43 | TCCTGAGCACTCACCCTTGA |  |  |
| DPA-44 | TGAGGTGACCGTGTTTCCCA |  |  |
| DPA-45 | TGCGTTCTGGCCATGACCCC |  |  |
| DPA-46 | TTTCCTTTGAGGCTCAGGGC |  |  |
| DPA-47 | TGCCGACTAGGCCCAGCACC |  |  |
| DPA-48 | TCAGCAGGAAAGCCAAGGAG |  |  |
| DPA-49 | TGAAGATGAGATGTTCTATG |  |  |
| DPA-50 | TGCTGAGTCTCCGAGGAGCT |  |  |
| DPA-51 | TGAGATGTTCTATGTGGATC |  |  |
| DPA-52 | TGGGAAACACGGTCACCTCA |  |  |
| DPA-53 | TGGAAGCTGTAATCTGTTCT |  |  |
| DPA-54 | TGGACAAGAAGGAGACCGTC |  |  |
| DPA-55 | TGCCCACGATGATGCCGACT |  |  |
| DPA-56 | TGGCCAAGCCTTTTCCTTTG |  |  |
| DPA-57 | GTGGCTGTGCAACGGGGAGC |  |  |
| DPA-58 | TCCCCTGGGCCCGGGGGTCA |  |  |
| DPA-59 | TCACCTCAGGGGGATCTGGA |  |  |
| DPA-60 | TCTCCTTCCAGATCCCCCTG |  |  |
| **CIITA** | | | |
| CIITA-01 | GCTGAACTGGTCGCAGTTGA |  |  |
| CIITA-02 | TGGAAGGTGATGAAGAGACC |  |  |
| **Control** | | | |
| Non-target | ACGGAGGCTAAGCGTCGCAA |  |  |

**Supplementary Table S3. Primer sequences for targeted deep sequencing.**

| Gene target | Primer forward | Primer reverse |
| --- | --- | --- |
| **B2M** | | |
| B2M-27-On | GATGAGTATGCCTGCCGTGT | ATGGGATGGGACTCATTCAG |
| B2M-27-Off-1 | TTTGAAAGGCACCCAAAGAG | TCTGCCCAGTCCTAAAGTGG |
| B2M-27-Off-2 | GCCTGTTACCCTCAGCTCAT | GGGAACTCCTTTTTGTGAACC |
| B2M-27-Off-3 | GTGGGGTTGCAGAATAAGGA | AATCAGAGGACCAACGTTCAA |
| B2M-27-Off-4 | CTGCTCTGGGGCCTGTATAA | CTCTGGAGCTCCCACATCAT |
| B2M-27-Off-5 | CTTTCCTGGGAGAGGAATAACA | CTTCACTCCCCATTTTATCAGG |
| **DRA** | | |
| DRA-18-On | CCGCAAGTTCCACTATCTCC | CCGAGTTTCACACAAGCATC |
| DRA-18-Off-1 | CAGAAAACGGTGTGCTTTGA | AGCAAGGTGCCTCTTAGTGC |
| DRA-18-Off-2 | TTTCAGGGTTGTGTGTGCAT | GAAAACTGAGGGGCTTATGTG |
| DRA-18-Off-3 | TCCCAGTTCTCCTCGTCCTA | GAGTGTGCCAGTGATGTTGG |
| DRA-18-Off-4 | TCCATTACCTGACCTTTGTGC | GGATGCCAGGAGATTATGGA |
| DRA-18-Off-5 | TGAGTTGGAGTTATAAAGAACGTCAT | ATGGCTGGAGTGCTTGACTT |
| **DQA** | | |
| DQA-40-On | TGCTCCACCTTGCAGTCATA | CCTGTGGTCAACATCACCTG |
| DQA-40-Off-1 | CTGAATCCCAGTTTTTCTGTCTTT | TCACTAACCATATTACCCTCAGCA |
| DQA-40-Off-2 | GGATTCACTGAGTCAAAAACACAC | CATAGTGCATGGCAGAGAGTAAGT |
| DQA-40-Off-3 | CATTGTCTGGATGAGAAAATTGAG | TTAACATGTGAGTGGAGAAAAGGA |
| DQA-40-Off-4 | AACTTGCATTTAGAGACAAGATGG | GCCACCACAACCCACTATTT |
| DQA-40-Off-5 | TGTGGTTTAAGAAAACTGGAAACA | TCCATCTCCAGCCAAAAGAT |
| **DPA** | | |
| DPA-13-On | ATGTGAACCACCCCATCACT | CGCCCTGAAGACAGAATGTT |
| DPA-13-Off-1 | ATGTTTCTTGCTGCCCAAAG | GCTGCCAATGACAGTGGTTA |
| DPA-13-Off-2 | ACTGCGTGTGGCTGAGAAG | CCGTGAACACAGACCTCACA |
| DPA-13-Off-3 | CCCCTCCTCTATTTTTCAGGA | CCAAAACCTGAACCAACCAA |
| DPA-13-Off-4 | CCGTCTGTAACAGGGTGAAG | AGCCCCTTGTGGGTACAAC |
| DPA-13-Off-5 | ACATGAGACACACCAGGCTTTA | GGGTAACATCTGACACCATCG |

**Supplementary Table S4. Primer sequences for chromosomal rearrangement detection.**

| **Primers** | **Sequence** |
| --- | --- |
| **B2M_forward** | CCCAAGTGAAATACCCTGGCA |
| **B2M_reverse** | AGCCCTTCCTACTAGCCTCA |
| **DRA_forward** | AATTTCTTGGGGAGGGGGTG |
| **DRA_reverse** | AGCTGGATAGTAGGAGAAGACAGT |
| **DQA1_forward** | GCATTCAGCAAATCCTTCTAGG |
| **DQA1_reverse** | CCCATGAAGTGTGGAAAACA |
| **DQA2_forward** | CCAAGTTTTACTCCCTTCTTCTCA |
| **DQA2_reverse** | AAACCCATGAAGTGGTGGAA |
| **DPA_forward** | TGTGAACTGGAGCTCTCTTGA |
| **DPA_reverse** | TATGAGGGCCAGAGGGAACAT |
| **HPRT_forward** | TGGGACTCTGTAGGGACCAG |
| **HPRT_reverse** | AGACAGAATGCTATGCAACCTT |
